# Exploring K2G30 Genome: A High Bacterial Cellulose Producing Strain in Glucose and Mannitol Based Media

**DOI:** 10.3389/fmicb.2019.00058

**Published:** 2019-01-30

**Authors:** Maria Gullo, Salvatore La China, Giulio Petroni, Simona Di Gregorio, Paolo Giudici

**Affiliations:** ^1^Department of Life Sciences, University of Modena and Reggio Emilia, Reggio Emilia, Italy; ^2^Department of Biology, University of Pisa, Pisa, Italy

**Keywords:** *Komagataeibacter xylinus*, bacterial cellulose, glucose, mannitol, xylitol, gluconic acid, genome sequencing

## Abstract

Demands for renewable and sustainable biopolymers have rapidly increased in the last decades along with environmental issues. In this context, bacterial cellulose, as renewable and biodegradable biopolymer has received considerable attention. Particularly, acetic acid bacteria of the *Komagataeibacter xylinus* species can produce bacterial cellulose from several carbon sources. To fully exploit metabolic potential of cellulose producing acetic acid bacteria, an understanding of the ability of producing bacterial cellulose from different carbon sources and the characterization of the genes involved in the synthesis is required. Here, K2G30 (UMCC 2756) was studied with respect to bacterial cellulose production in mannitol, xylitol and glucose media. Moreover, the draft genome sequence with a focus on cellulose related genes was produced. A pH reduction and gluconic acid formation was observed in glucose medium which allowed to produce 6.14 ± 0.02 g/L of bacterial cellulose; the highest bacterial cellulose production obtained was in 1.5% (w/v) mannitol medium (8.77 ± 0.04 g/L), while xylitol provided the lowest (1.35 ± 0.05 g/L) yield. Genomic analysis of K2G30 revealed a peculiar gene sets of cellulose synthase; three *bcs* operons and a fourth copy of *bcsAB* gene, that encodes the catalytic core of cellulose synthase. These features can explain the high amount of bacterial cellulose produced by K2G30 strain. Results of this study provide valuable information to industrially exploit acetic acid bacteria in producing bacterial cellulose from different carbon sources including vegetable waste feedstocks containing mannitol.

## Introduction

Demands for renewable and sustainable biopolymers have rapidly increased in the last decades along with environmental issues. In this context, bacterial cellulose (BC), as biocompatible, renewable and biodegradable biopolymer has received considerable attention.

The primary structure of BC consists of a β-1,4-glucan chain which undergo aggregation events to form a ribbon-like structure (Saxena et al., 1994; Brown, 1996). These ribbons form the secondary structure that generate the well-structured 3D network, characteristic of BC (Koyama et al., 1997). The tertiary structure, as a result of intermolecular hydrogen bonds and van der Waals forces, stabilizes the entire structure through an intramolecular hydrogen bond network by hydroxyls and ring oxygen among glucose residues. Each repeating unit has a directional chemical asymmetry with respect to its molecular axis (a hemiacetal unit and hydroxyl group) (Koyama et al., 1997). The fibrous network forms a hydrogel film at air surface of culture media.

Differently from its equivalent produced by plants, BC does not contain lignin and hemicellulose, it has higher degrees of purity, polymerization, crystallinity, tensile strength, water holding capacity, and biological adaptability (Costa et al., 2017; Gullo et al., 2018). Structurally, BC differs from plant cellulose on the basis of cellulose I_α_ and I_β_ content (Atalla and Vanderhart, 1984). These two allomorphs coexist always in nature and differ for the crystal system: the I_α_ is characterized by triclinic system, whereas I_β_ by a monoclinic system. Cellulose I_α_ is the predominant allomorph in BC, ranged from 60 to 80%. On the other hand, the allormorph I_β_ is the predominant form of plant cellulose (Nishiyama et al., 2002, 2003).

All BC properties are strictly linked to both intracellular biosynthesis and extracellular self-assembling mechanism. It is widely accepted that BC is synthesized within the bacterial cell as individual molecules. The biosynthesis occurs on the periplasmic space by cellulose synthase (CS), a membrane protein complex formed by a series of subunits: BcsA, BcsB, BcsC, and BcsD, of which BcsA and BcsB represent the catalytic core of CS (Gullo et al., 2018). The CS genes are organized in an operon that, based on the genus, are divided into three classes, which differ in content and structure. The first class is represented by the operon originally described in the *Komagataeibacter xylinus* species. The second one, described for the first time in *E. coli*, contains also a divergent locus, which includes genes involved in natural BC modification (Thongsomboon et al., 2018). Finally, the third class of BC operon, described in *A. tumefaciens*, consists of two convergent operons. The first three genes are ortholog of *bcsA, bcsB*, and *bcsZ* from *K. xylinus*, while the others are typical of *A. tumefaciens* (Matthysse et al., 2005). Similar operons are also found in members of *Actinobacteria* and *Firmicutes phyla* (Römling and Galperin, 2015).

As material generally recognized as safe (GRAS) by the United States Food and drug administration (FDA) in 1992, BC can be utilized as a fiber for different applications in biomedical, cosmetics and food (Shi et al., 2014). Main biomedical uses include supports as substitute artificial skin, hemostatic materials, wound healing scaffolds, and controlled drug delivery (Pavaloiu et al., 2014; Picheth et al., 2017). Recently very interestingly insights were obtained using BC as a biocarrier of dihydroxyacetone, in masking the symptoms of vitiligo by providing skin pigmentation (Stasiak and Płoska, 2018).

In cosmetic field, BC is an ingredient for facial mask creams and as a powder in facial scrubs products in association with other natural materials (as olive oil, Vitamin C, *Aloe vera* extract, and powdered glutinous rice).

In food, BC can be used as dietary fiber and as adjuvant thanks to the ability to acquire flavors and colors. It occurs in the manufacturing of nata de coco, a Philippine dessert produced from fermented coconut water, and in Kombucha tea, a fermented beverage obtained from alcoholic and acetic fermentation of sugared tea (Gullo et al., 2018).

BC production was described for different bacterial species, comprising *Rhizobium leguminosarum, Burkholderia* spp., *Pseudomonas putida, Dickeya dadantii, Erwinia chrysanthemi, Agrobacterium tumefaciens, Escherichia coli*, and *Salmonella enterica* species (Chawla et al., 2009; Jahn et al., 2011). Within acetic acid bacteria (AAB), different genera were reported as BC producers including *Acetobacter, Gluconacetobacter* and *Komagataeibacter* (Mamlouk and Gullo, 2013). AAB are considered a very versatile group of bacteria involved in a wide range of industrial process for the production of different compounds, such as acetic acid in vinegar production, gluconic acid, 2-keto-L-gluconic acid, 5-keto-L-gluconic acid, 2-keto-gulonic acid, and dihydroxyacetone (Stasiak and Blazejak, 2009; La China et al., 2018). In vinegar production, other than acetic acid they can also form BC which is considered as a disadvantage because it negatively affects the process and the sensorial properties of the product (Gullo and Giudici, 2008; Gullo et al., 2016). On the other hands, vinegar has been used as an appropriate substrate for studying the mechanism of BC synthesis by AAB (Gullo et al., 2018). Species of the genus *Komagataeibacter* are widely detected in vinegar such as *K. europaeus* and its closely related species *K. xylinus*, which is considered as a model organism for BC synthesis (Römling, 2002).

Given the wide range of use of BC and the increasing of the market of bacteria cellulose-based materials, which is expected to exceed 500 million US dollars by 2023 (based on market research report^1^), there is a need to link the knowledge of science to its industrial scale-up. Although a number of studies have highlighted great potential of application, others demonstrate limitations in term of process and economic sustainability (Gullo et al., 2017; Islam et al., 2017; Basu et al., 2018).

Main issues in BC production arise from the organism and the cultivation conditions, which affect the implementation of advantageous industrial processes. However, a number of works aiming at selecting robust wild strains and obtaining engineered strains are available. These studies mainly focuses *Komagataeibacter* species (*K. xylinus* and *K. hansenii*), tested in different culture conditions (Hwang et al., 1999; Kuo et al., 2010; Costa et al., 2017; Gullo et al., 2017). The most widely system of production is the static regime by which layers of different form and thickness are obtained, according to the ratio surface/volume (S/V) of vessels. Also the production by agitated cultivation system is reported, but it seems to provide a lower yield and generally BC is formed as spheres (Gullo et al., 2018).

Regarding the raw materials for producing BC, both the need to reduce costs and the need to provide more sustainable productions, encouraged the use of vegetable waste feedstocks containing suitable carbon sources (Kuo et al., 2010; Cheng et al., 2017; Costa et al., 2017). Promising results were provided from lignocellulosic materials such as wheat straw, sugarcane bagasse, rice straw, hydrolysate fiber sludge, and corn steep liquor (Hwang et al., 1999; da Silva Filho et al., 2006; Cavka et al., 2013; Narh et al., 2018). Carbon sources mainly contained in these products are glucose, sucrose, and polyols as mannitol and xylitol, that are also naturally found in fruits, other vegetables, and also produced by bacteria and yeasts (Song and Vieille, 2009).

Focusing, on genes related to BC synthesis, in this work we present the genome sequencing of K2G30 (UMCC 2756), an AAB strain from Unimore Microbial Culture collection, previously selected as highly BC producing strain (Gullo et al., 2017). We also tested the BC production ability of K2G30 in two alternative carbon sources (mannitol and xylitol), which usually occur in waste vegetables.

## Materials and Methods

### Bacterial Strain and Culture Conditions

K2G30 strain used in this study was previously isolated from pellicle fraction of Kombucha tea (Mamlouk, 2012) and safely deposited at the Unimore Microbial Culture Collection (UMCC) under the collection number UMCC 2756. The strain was cultivated in aerobic conditions at 28°C in GY broth (glucose 5.0% w/v and yeast extract 1.0% w/v; pH 4.46 (at time 0 of cultivation), when appropriate, agar (0.8% w/v) was supplemented. Bacterial cellulose was produced in GY, mannitol medium (1.5% mannitol (w/v), 2% yeast extract (w/v) and 0.5% polypeptone (w/v); pH 5.75 at time 0 of cultivation) (Oikawa et al., 1995b) and xylitol medium (5% xylitol (w/v), 1% yeast extract (w/v) and 1% tryptone (w/v); pH 5.65 at time 0 of cultivation) (Singhsa et al., 2018). Glucose, mannitol and xylitol were purchased from Merck KGaA, Darmstadt, Germany; yeast extract from Thermo Fisher Scientific Inc., All the media were sterilized by autoclaving at 121°C for 15 min prior the use.

### Bacterial Cellulose Production in Glucose, Mannitol, and Xylitol Broths

K2G30 was first cultivated in GY broth at 28°C for 5 days under static conditions. Aliquots were used to prepare preinocula in mannitol and xylitol broths, respectively. Triplicate assays were conducted in 500 mL beakers (diameter 8.7 cm) containing 150 mL of the respective broths inoculated with 5% (v/v) of the preinoculum culture. Cultures were incubated at 28°C for 9 days under static conditions. Moreover, cultivation in agitated conditions (130 rpm/28°C/5 days) was performed in 100 mL flasks using 40 mL of each broth inoculated with 5% (v/v) of the preinoculum.

### Bacterial Cellulose Harvesting, Purification, and Weighing

BC pellicles collected at 3, 6, and 9 days of cultivation were washed with deionized water and treated with NaOH 1 M at 80°C for 40 min to remove bacterial cells; they were washed several times with deionized water to reach neutral pH, and finally dried at 37°C until constant weight. After perming experiments in triplicate, meaningful values were determined. The BC weight was expressed as previously reported (Gullo et al., 2017).

### Analytic Determinations

D-glucose and D-Gluconic acid were measured by enzymatic kits (Megazyme Ltd., Bray, Ireland) according to the manufacturer’s instructions. pH was measured using an automatic titrator (TitroLine^®^EASY SCHOTT Instruments GmbH, Mainz, Germany), equipped with an SI Analytics electrode (SI Analytics, GmbH, Mainz, Germany). The samples were obtained collecting 5 mL of each culture medium in three-day intervals.

### Genomic DNA Extraction and Sequencing

Genomic DNA (gDNA) extraction from K2G30 was performed on a total 40 ml of GY culture, after 5 days of incubation at 28°C. The liquid culture was centrifuged at 10,000 × g/4°C/10 min. gDNA was extracted as previously described (Gullo et al., 2006) and it was quantified using Qubit 2.0 (Invitrogen, Carlsbad, CA, United States). A total 100 ng was used for sequencing. The whole genome of *K. xylinus* K2G30 was sequenced by Admera Health LCC (South Plainfield, NJ, United States) using Nextera XT DNA Sample Preparation Kits (Illumina) and sequenced using the Illumina HiSeq X.

### *De novo* Genome Assembly and Annotation

The primary quality check was performed using FastQC and Trimmomatic v0.36 tool (Bolger et al., 2014) which was used to remove bases with a Phred score <20. Spades v1.10.1 (Bankevich et al., 2012) was used for *de novo* genome assembly using careful option and kmer size of 21, 33, and 55. The quality of consensus sequences was evaluated using Quast v4.5 (Gurevich et al., 2013) and reads with length lesser than 1 Kbp were discarded. Resulted contigs were used for genome annotation. Putative coding regions were identified by Prodigal v2.6.3 (Hyatt et al., 2010), while tRNA and rRNA were predicted using tRNAscan-SE v1.3.1 and RNAmmer (Lowe and Eddy, 1997; Lagesen et al., 2007). Functional annotation of translated coding regions was performed using Blastp v2.7.1 (McGinnis and Madden, 2004) against NCBI non-redundant database and Uniprot, setting E-value threshold as 1E-5. Hmmscan v3.1b2 (Mistry et al., 2013) was used for protein domain annotation via Pfam database and protein family definitions via Tigrfam databases. Cluster of orthologous groups (COG) were retrieved using EggNog-mapper v1.0.3 (Huerta-Cepas et al., 2017). These data sources were combined and manually curated using Arthemis genome browser (Carver et al., 2012) to assert a product description for each predicted protein. Genome ideogram reconstruction was performed using Circos v0.69 (Krzywinski et al., 2009). The genome version discussed in this study is the version K2G30_v1.0. Fastq files and genome assembly fasta file are available at GeneBank, under the accession number QQBI00000000.

### Phylogenetic Genome Reconstruction

16S and 23S rRNA sequences and genome-to-genome sequence similarity analysis were performed. The related 16S and 23S rRNA sequences were downloaded from Silva SSU and LSU databases and from Joint Genome Institute (JGI) using IMG. Both 16S and 23S were separately aligned using Muscle aligner and then concatenated using MEGAX (Edgar, 2004; Kumar et al., 2018). From concatenated alignment, a maximum likelyhood phylogenetic tree was generated using Tamura-Nei model (Tamura and Nei, 1993) with 1000 replicated, setting gamma distribution option. We used also alternative method to recognize K2G30 phylogeny, as digital DNA-DNA hybridization analysis. A total of 19 genomes of *Komagataeibacter* genus (taxid 1434011) retrieved from NCBI were used for analysis. The average nucleotide identity values using BLAST method (ANIb) and tetranucleotide usage patterns (TETRA) for all pairwise comparisons of 19 *Komagataeibacter* genomes were determined. ANIb and TETRA were calculated by the use the pyani module (Pritchard et al., 2016).

## Results and Discussion

### K2G30 Genome Features and Bacterial Cellulose Synthase Genes

**FIGURE 1:**
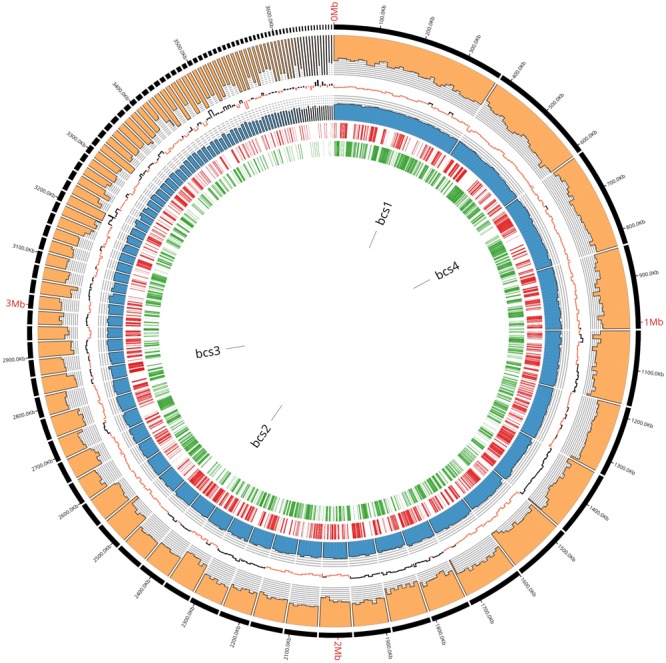
K2G30 genome circular representation. From outside to inside: contigs with relative lenghts expressed in bp; coverage; GC skew expressed in positive changes in GC content (black) and negative (red); GC content expressed in percentage; forward CDS; reverse CDS; bcs operons genome localization.

In order to describe the genetic organization of BC production related genes, the genome of K2G30 was sequenced, annotated and explored. The genome of K2G30 consists of 101 contigs with a total length of 3.63 Mbp and a coverage average of 700X (Figure 1). A total of 22 contigs were more or equal than 50 Kbp in size and N50 and L50 were 86.67 Kbp and 11, respectively. The G + C content was 62.77%, which is near to the median G + C content in sequenced genomes of *K. xylinus* strains.

One operon of rRNA genes was retrieved including one copy of 5S, 16S, and 23S, respectively. The number of predicted coding DNA sequences (CDS) were 3380. The genome properties and statistics are summarized in Table 1. More than 75% of CDS were assigned to cluster of orthologous-group classification and functional categories (COG) (Table 2).

**Table 1:** K2G30 Genome features.

Properties	Value
Contigs	101
Lenght	3.63 Mbp
N50	86.67 Kbp
L50	11
Depth of coverage	700X
CDS	3380
rRNA	3
tRNA	51
tmRNA	1
Uniprot	64.70%
Pfam	87.66%
COG	78.04%
KEGG	50.5%

**Table 2:** Genome cluster of orthologous-group classification and functional categories.

Categories	Functional group	Percentage (%)
C	Energy production and conversion	6.55
D	Cell cycle control, cell division, chromosome partitioning	0.96
E	Amino acid transport and metabolism	7.34
F	Nucleotide transport and metabolism	2.88
G	Carbohydrate transport and metabolism	5.31
H	Coenzyme transport and metabolism	3.81
I	Lipid transport and metabolism	2.28
J	Translation, ribosomal structure and biogenesis	5.31
K	Transcription	6.2
L	Replication, recombination and repair	7.55
M	Cell wall/membrane/envelope biogenesis	6.27
N	Cell motility	0.14
O	Posttranslational modification, protein turnover, chaperones	3.81
P	Inorganic ion transport and metabolism	6.98
Q	Secondary metabolites biosynthesis, transport and catabolism	1.17
S	Function unknown	26.81
T	Signal transduction mechanisms	2.67
U	Intracellular trafficking, secretion, and vesicular transport	2.49
V	Defense mechanisms	1.46

**FIGURE 2:**
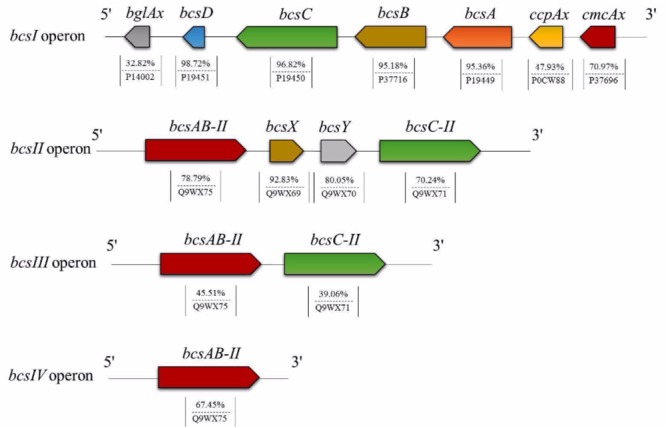
The arrangement of *bcs* operons and BC related genes organization in K2G30.

Based on Uniprot alignment, in K2G30 genome, three copies of *bcs* operons were annotated, of which only one (*bcs1)* contains the full enzymatic set of CS related genes (BcsA, BcsB, BcsC, and BcsD) and the accessory proteins CMCax, ccpAx, and BglAx (Figure 2). *Bcs*1 operon was localized in contig 1, at the genomic position 256838-270633 bp. The full set of CS genes is represented by *bcsA, bcsB* (the catalytic core of CS), *bcsC, bcsD*, preceded by *cmcax*, and *ccpax* genes, in upstream position, while in downstream position *bglAx* was found. The sequences similarity span from 32.82% to 98.72% with and minimum e-value score of 2e^-19^.

*Cmcax* gene encodes for endo-β-1,4-glucanase, while the *ccpax* role is not clear. However, it was hypothesized that it can be involved in the structural organization of the terminal complex, cooperating with BcsD (Sunagawa et al., 2013). The downstream *bglax* gene encodes for β-glucosidase and seems to have also the glucosyltransferase activity (Tajima et al., 2001).

A second *bcs* operon (*bcs*2) was found in contig 19, located from 5579 to 16126 bp. This operon displays the classical architecture of *bcs2* described in other *Komagataeibacter* strains (Römling and Galperin, 2015). Differently from *bcs1* operon, in *bcs*2, the genes that encode for BcsA and BcsB subunits are fused, named *bcsAB.* No gene codifying for BcsD was detected, while the gene encoding for BcsC was detached from *bcsAB* by other two genes (*bcsX* and *bcsY*), generally described as peculiar in *bcs2* operon. The sequence similarity based on aminoacids sequences alignment was 78.79% for *bcsAB* (e-value of 0.0), instead for *bcsC* the sequence similarity was 70.24% (e-value of 0.0). The similarity of *bcsX* and *bcsY* were 92.83% and 80.05%, respectively.

*bcs3* operon is located in contig 28 at position 28895-37149 bp and contains genes that encode for BcsA, BcsB and BcsC subunits. Also in this operon, the genes that codify for BcsA and BcsB were fused. In addition, in K2G30, a fourth copy of *bcsAB* genes was retrieved in contig 3, having an aminoacid sequence similarity of 67.45% (e-value score of 0.0). The high number of CS catalytic subunits can explain the high BC yield previously obtained from K2G30 (23 g/L) (Gullo et al., 2017). Only in another genome of *K. xylinus* species the fourth copy of *bcsAB* gene was described (Liu et al., 2018). Moreover, a variable copy number of *bcs* operon was previously reported for cellulose-producing AAB (Gullo et al., 2018). *K. hansenii* species were described to contain three copies of *bcs* operon (Iyer et al., 2010; Florea et al., 2016). In *K. xylinus*, the presence of one *bcs* operon was reported for a not BC producer strain (NBRC 3288) (Ogino et al., 2011), whereas two copies were retrieved in *K. xylinus* E25 (Kubiak et al., 2014).

### Phylogenetic Analysis

Individual alignment of 16S and 23S rRNA genes of 19 *Komagataeibacter* species were checked manually and clipped at the same length. A phylogenetic tree was generated from concatenated rRNA genes with a total length of 3687 nucleotides (nt): 16S (1118 nt) and 23S (2569 nt).

**FIGURE 3:**
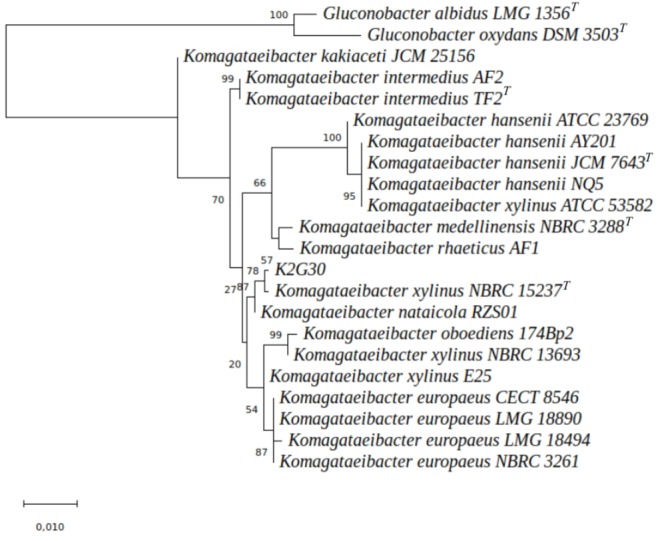
ML tree representing the phylogenetic distances among 19 *Komagataeibacter* genomes. The node numbers indicate the bootstrap values. The branch length was expressed in 0.010 unit.

The rRNA genes of two species of *Gluconobacter* genus (*G. albidus* LMG 1356^T^ and *G. oxydans* DSM 3503^T^), were included in the dataset and used as outgroup. The ML tree (Figure 3) displays that *Komagataeibacter* species clustered in three major groups, the *K. hansenii*, the *K. europaeus* and the *K. intermedius* species. *K. medellinensis* and *K. rhaeticus* were closed in a single clade. K2G30 was clustered with the type strain (*K. xylinus* NBRC 15237^T^) and *K. nataicola* RZS01, with a bootstrap percentage of 78%. The information gained from the phylogenetic analysis provides suitable depiction of the evolutionary position of K2G30 strain, but does not translate directly into the overall similarity of the genomes. Here, we used two approaches of digital DNA-DNA hybridization, ANIb and TETRA, that are considered two of the traditional “gold standard” for circumscribing bacterial species (Teeling et al., 2004; Konstantinidis and Tiedje, 2005). The required threshold to ascribe one species using ANIb (94% of genome sequence similarity) and TETRA (0.997) were previously defined (Richter and Rosselló-Móra, 2009).

**FIGURE 4:**
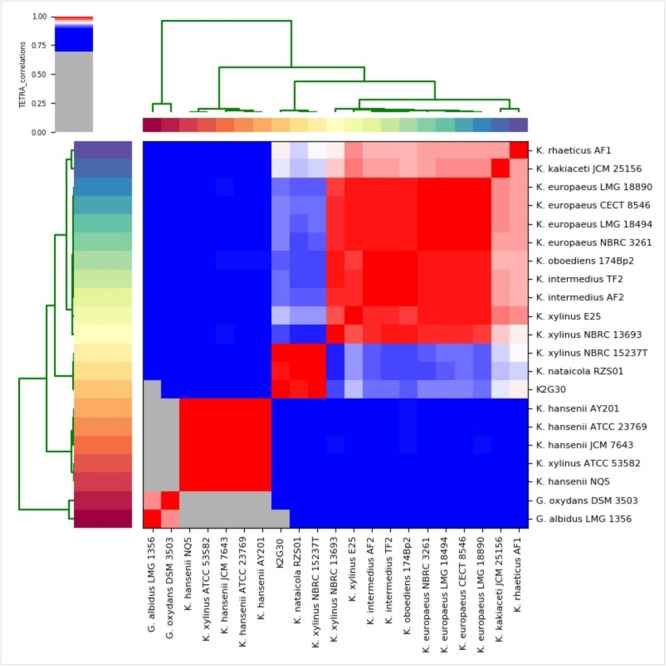
TETRA heatmap of 21 *Komagataeibacter* genomes sequences (derived from Supplementary Table S2). TETRA values are represented in the central bi-color gradient heatmap (red gradients ≥ 96%; white = 95%; blue gradients ≤ 94%).

We used the genome sequences of the species considered for ML tree reconstruction to produce the ANIb and TETRA distance matrices. As shown in Supplementary Table S1, the genome sequence similarity from the comparison of K2G30 and *K. xylinus* NBRC 15237^T^ was 93.38%, just below the minimum threshold required to define a bacterial species. ANIb heatmap (Supplementary Figure S1) confirms the clustering order of the phylogenetic tree (Figure 3), showing the same three clades. The data from TETRA heatmap (Figure 4) were congruent with ANIb analysis and the ML tree, showing the same three large clades. The TETRA correlation value between K2G30 and *K. xylinus* NBRC 15237^T^ was 0.9973, while the correlation value for the pairwise K2G30 and *K. nataicola* RSZ01 is 0.9952, lower than the threshold.

It is clear from literature and from Figure 3, 4 and Supplementary Figure S1 of this study, that several strains attributed to the species *K. xylinus* do not associate with the type strain of the species (NBRC 15237^T^), but they cluster with other species of the genus (Lisdiyanti et al., 2006). Due to these misinterpretations, a critical revision of *K xylinus* strains published and available in public culture collections is advisable.

Interestingly, in our analysis in which only sequenced genomes of *Komagataeibacter* are represented, just the strain we characterized (K2G30), clusters with *K. xylinus* type strain NBRC 15237^T^ and could be confidently attributed to this species. For all those strains reported in literature as high BC producers that have been attributed *K. xylinus* species in the absence of genomic data, a critical re-examination and an identification using genomic data is needed. This would allow to verify if high BC production is indeed a specificity of *K. xylinus* or also of other species within the genus, which in former years and in absence of appropriate resolution tools, have been attributed to *K. xylinus*, but are indeed representatives of different species.

### Glucose, Mannitol, and Xylitol as Carbon Sources for Producing Bacterial Cellulose

**FIGURE 5:**
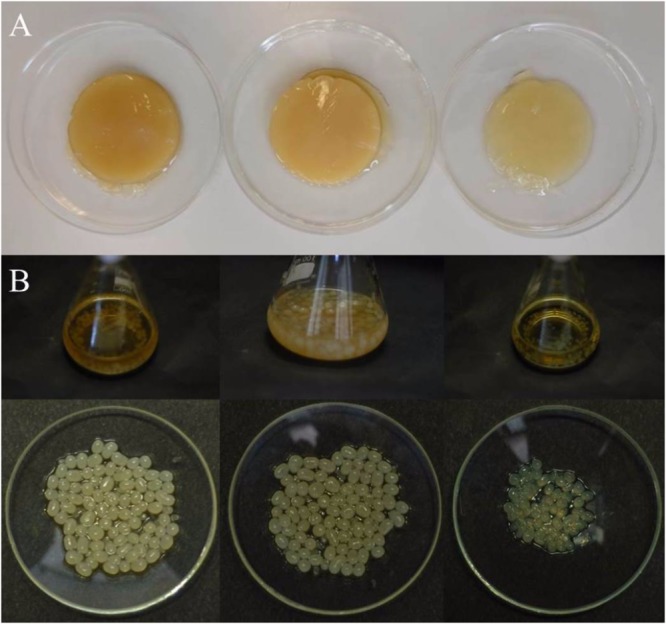
BC produced by K2G30 in static **(A)** and agitated **(B)** conditions. From left to right: BC produced in mannitol, glucose, and xylitol, respectively.

With the aim to select the best cultivation system for obtaining BC pellicles from the strain K2G30, cultures were grown on GY broth, in both static and agitated conditions, and on GY agar medium. Well compacted BC membranes were obtained in static cultivation, whereas spheres of different size were produced in agitated cultivation system (Figure 5A,B). When we cultivated K2G30 on GY agar medium, we did not observe single colonies but a soft layer on the surface of the plates. An increase of BC yield has been obtained by different strategies including the use of double carbon sources e.g., glucose and ethanol, modified sugars and polyols (Oikawa et al., 1995a,b; Canilha et al., 2008; Singhsa et al., 2018).

In this study, BC production by K2G30 was tested using two polyols (mannitol and xylitol), as alternative carbon sources to glucose, by static cultivation system at 28°C for a total of 9 days, according to Oikawa et al., 1995b and Singhsa et al., 2018, respectively. Assays were performed in vessels (500 mL beakers) having a S/V ratio of 0.40 cm^-1^. BC pellicles were visible on the surface of all performed trials within 2 days of cultivation. However, different BC yields were reached in glucose broth (reference medium), mannitol and xylitol broths. The highest BC yield was observed in mannitol (1.5% (w/v) initial mannitol content) whereas xylitol broth (5% (w/v) initial xylitol content) was less performing in BC synthesis (Table 3). Within strains of the species *K. xylinus* the ability to produce BC from xylitol could be a variable traits, as previously observed by Singhsa et al., 2018, which tested five strains that produced low or moderate amount of BC, except for one strain that produced BC in higher amount (more than 1.0 g/L during 7 days cultivation).

**Table 3:** Bacterial cellulose production and pH values (S/V of vessel 0.40 cm^-1^)^1^.

	Glucose (5% w/v)		Mannitol (1.5 w/v)		Xylitol (5% w/v)	
Time (days)	BC (g/L)	pH	BC (g/L)	pH	BC (g/L)	pH
0	0.0	4.46 ± 0.05	0	5.75 ± 0.03	0	5.65 ± 0.39
3	1.149 ± 0.001	3.81 ± 0.09	0.188 ± 0.003	5.71 ± 0.09	0.048 ± 0.001	5.57 ± 0.02
6	3.805 ± 0.001	3.24 ± 0.03	2.368 ± 0.001	5.39 ± 0.04	0.527 ± 0.001	5.51 ± 0.03
9	6.167 ± 0.024	3.10 ± 0.02	8.766 ± 0.043	5.58 ± 0.83	1.356 ± 0.050	5.23 ± 0.08

*^1^The data are averages based on three trials, reporting the standard error values.*

In glucose broth (5% w/v of initial glucose content), the BC yield obtained was considerable, but less compared to the amount produced in mannitol broth (Table 3). From these results, it was shown that using mannitol the BC yield was increased of 43% than using glucose and that xylitol was not a preferred carbon sources of K2G30 for producing BC.

In the BC production process, the accumulation of organic acids in the culture broth, such gluconic acid and acetic acid, due to the oxidative metabolism of AAB, induces pH decreases far below the optimal value for BC production, that is estimated higher than 4 (Gullo and Giudici, 2008; Giudici et al., 2016). As previously reported the pH reduction at values below 4 is suboptimal for BC synthesis (Jonas and Farah, 1998; Gullo et al., 2017). In this study, a negligible production of gluconic acid, which induced a limited pH reduction, was observed both in mannitol and xylitol broths; whereas in glucose broth, BC formation was followed by glucose oxidation to gluconic acid and a pH decrease below 4 (Table 4).

**Table 4:** Gluconic acid (GlcA) production and pH values during BC production in glucose, mannitol and xylitol media (S/V of vessel 0.40 cm^-1^)^1^.

	Glucose		Mannitol		Xylitol	
Time (days)	GlcA (g/L)	pH	GlcA (g/L)	pH	GlcA (g/L)	pH
0	1.353 ± 0.741	4.46 ± 0.05	0.927 ± 0.017	5.75 ± 0.03	0.530 ± 0.044	5.65 ± 0.39
3	12.887 ± 0.027	3.81 ± 0.09	1.121 ± 0.021	5.71 ± 0.09	1.182 ± 0.029	5.57 ± 0.02
6	19.580 ± 0.091	3.24 ± 0.03	1.109 ± 0.033	5.39 ± 0.04	1.144 ± 0.028	5.51 ± 0.03
9	29.779 ± 0.046	3.10 ± 0.02	1.243 ± 0.014	5.58 ± 0.83	1.193 ± 0.060	5.23 ± 0.08

*^1^The data are averages based on three trials, reporting the standard error values.*

As in mannitol, also in xylitol broth the gluconic acid production was very low. This can be explained considering pentose and glucuronate metabolism (pathway ID KO00040), in which through different steps xylitol is converted into UDP-glucose, the precursor of BC.

Considering the metabolic pathways of mannitol and xylitol based on Kegg pathways maps, D-mannitol was converted to D-fructose and used to produce BC via galactose metabolism (pathway ID KO00051-KO00052). The candidate enzyme for this conversion seems to be D-mannitol dehydrogenase (EC 1.1.1.67). Differently from the membrane-bound enzyme polyol dehydrogenase (EC 1.1.5.2) that has low substrate specificity, D-mannitol dehydrogenase is a soluble enzyme characterized by high specificity for its substrate (Oikawa et al., 1997).

Contrary to D-mannitol, the production of BC from xylitol is a more complex pathway, involving several steps, which result less advantageous energetically for the cell.

## Conclusion

In the present study, K2G30 genome was sequenced and annotated in order to describe the key gene sets involved in BC synthesis. From genome analysis, four copies of the enzymatic core of CS and three copies of *bcs* operons, were retrieved, explaining the high BC yield obtained from K2G30. From phylogenetic analysis, also the need of a re-examination within the *Komagataeibacter* genus was emphasized.

K2G30 is able to grow and to produce BC using different carbon sources, this is an additional attribute that highlights the versatility of the strain.

The choice of the carbon sources is one of the most important point in BC production, particularly for the obtainable yields. Effective BC production from glucose is questionable, due to the oxidative metabolism leading to gluconic acid production and concomitant pH decrease. Our evidences confirm the limit of using glucose as a carbon source and the suitability of other carbon sources, like mannitol, by which gluconic acid production is negligible. Considering that for industrial BC production, raw material remains a weighty production cost, the evaluation of waste feedstocks is a challenge. Since mannitol is found in a number of vegetal wastes, their use in producing BC could be promising. We therefore proposed a strategy that integrate information deriving from technological and genomic data as a platform for selecting strains and for optimizing bioprocessing for BC large-scale production.

## Author Contributions

MG designed the research, supervised the work and wrote the manuscript. SL performed laboratory experiments, bioinformatic analysis, and participated in writing the manuscript. GP drafted the work and critically revised the manuscript. SD reviewed and edited the manuscript. PG contributed to the interpretation of data and critically revised the manuscript. All the authors read and approved the manuscript.

## Conflict of Interest Statement

The authors declare that the research was conducted in the absence of any commercial or financial relationships that could be construed as a potential conflict of interest.

## Footnotes

1 https://www.marketresearchreports.com

## Supplementary Material

FIGURE S1 ANIb heatmap of 21 *Komagataeibacter* genomes sequences (derived from Supplementary Table S1). ANIb values are represented in the central bi-color gradient heatmap (red gradients ≥ 96%; white = 95%; blue gradients ≤ 94%).

TABLE S1 ANIb pairwise similarity values for all *Komagataeibacter* genome sequences (n = 21).

TABLE S2 TETRA pairwise similarity values for all *Komagataeibacter* genome sequences (n = 21).
