# Supplementary figures and images for: Exploring K2G30 Genome: A High Bacterial Cellulose Producing Strain in Glucose and Mannitol Based Media
Source: Front Microbiol. 2019 Jan 30;10:58. doi: 10.3389/fmicb.2019.00058 (PMC6363697; doi:10.3389/fmicb.2019.00058)

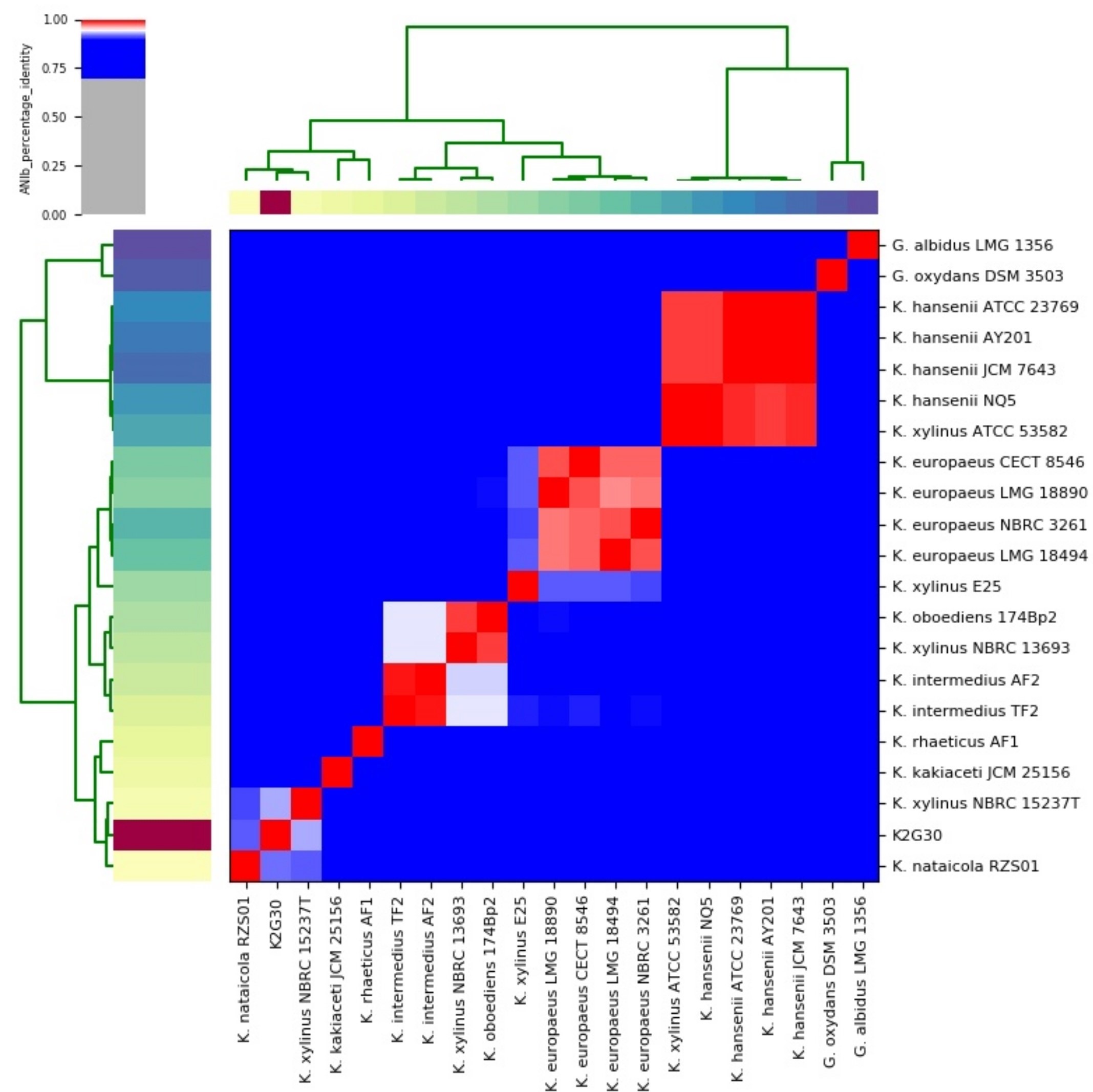

Supplement: FIGURE S1 — ANIb heatmap of 21 Komagataeibacter genomes sequences (derived from Supplementary Table S1). ANIb values are represented in the central bi-color gradient heatmap (red gradients ≥ 96%; white = 95%; blue gradients ≤ 94%). [file Image_1.JPEG]
